# Acknowledgment of *Ad Hoc* Reviewers

**DOI:** 10.1128/mSystems.00797-19

**Published:** 2019-12-17

**Authors:** Jack A. Gilbert

**Affiliations:** a University of California, San Diego, San Diego, California, USA

## EDITORIAL

In 2019, we have seen *mSystems* become one of the premier destinations for microbiome and systems biology research, especially with a push into synthetic microbiology. Without reviewers dedicating their expertise and time to supporting the timely peer review of these studies, we would not be successful in creating a venue that publishes the highest-quality scientific literature. Solid peer review creates trust that the work published in *mSystems* is reliable. However, it is also fun to remember that like pedestrians, cyclists, and motorists, who can all switch places at some point and yet fail to act with sufficient empathy toward the other two, reviewers, authors, and editors of society journals often need to remember that the service that they provide will reflect on the service that they receive. So thank you to all the reviewers for doing unto others as you would have done unto thyself! Thank you also for supporting open access publishing, and we look forward to exploring the power of open peer review in 2020.

Frank M. Aarestrup
Sophie Saphia Abby
Mark D. Adams
Joshua N. Adkins
Anton Aebischer
Ameeta K. Agarwal
Masato Akiba
Alexander Aksenov
Luis D. Alcaraz
Nezar Al-Hebshi
Caitilyn Allen
Jacob Allen
Emma Allen-Vercoe
João M. G. C. F. Almeida
Hasan Ali Al-Talhi
Stefano Amalfitano
Katherine R. Amato
Namasivayam Ambalavanan
Amnon Amir
Alongkorn Amnuaykanjanasin
Karthik Anantharaman
Tessa Anderman
Brooke Anderson
Christopher Joseph Anderson
Rika Anderson
Andrés Andrade-Domínguez
Haike Antelmann
Josefa Antón
Rafael Araos
Ana Paula Arez
Hector Arguello
Anna Arís
E. Virginia Armbrust
Kristine Arnvig
Mario Arrieta-Ortiz
Bernard P. Arulanandam
Alexander Askenov
Marina Elisabeth Aspholm
Louis Ates
Frank O. Aylward
Ramy K. Aziz
Giovanni Bacci
Brett J. Baker
Katherine Barbeau
Sonia L. Bardy
Mohammed Barigou
Tyler Barnum
Lars Barquist
Dariusz Bartosik
Jose M. Bautista
Barbara Bayer
Michael Baym
Wendy Bedale
Vivian Bellofatto
Ronen Ben-Ami
Sarah Ben Maamar
Richard J. Bennett
Kathryn Bernard
Hans C. Bernstein
Claire Bertelli
Stefan Bertilsson
Cora Betsinger
Rodrigo Carvalho Bicalho
Steven Biller
Emanuele G. Biondi
Wilbert Bitter
Linda Blackall
Jeffrey Blanchard
Jill R. Blankenship
Louis-Marie Bobay
Rich Boden
Nicholas Andrew Bokulich
Jennifer M. Bomberger
Elizaveta A. Bonch-Osmolovskaya
Joe Bondy-Denomy
Rafaella C. Bonugli-Santos
Emanuele Bosi
Germán Bou
Sebastien Boutin
Robert Bowers
John Dallas Boyce
Nanette R. Boyle
Joel Bozue
Gerrit Brandis
Kristoffer Brandvold
Asker Daniel Brejnrod
Patrizia Brigidi
Ilana Lauren Brito
Robert A. Britton
Nichole A. Broderick
Dennis Brown
Pamela J. B. Brown
Silvio D. Brugger
Jennifer Brum
Donald A. Bryant
Samuel Bryson
Dongbo Bu
Alison Buchan
Silvia Bulgheresi
Lorinda Bullington
Joy Buongiorno
Mariana X. Byndloss
Léa Cabrol
Michelle C. Callegan
Antonio Camargo
Valerie Jean Carabetta
Kyle Card
Maureen A. Carey
Erin Carlson
Alex Carr
Stephanie A. Carr
Víctor J. Carrion
Eric Cascales
Grayson L. Chadwick
Baofeng Chai
Josephine R. Chandler
Yanjie Chao
Trevor Charles
Aleksandra Checinska
Casey Chen
Liang Chen
Tingtao Chen
Wei Chen
Xingqun Cheng
Yin-Ru Chiang
Siok-Fong Chin
Barbara Chirullo
Ludmila Chistoserdova
Byung-Kwan Cho
Hana Cipcic Paljetak
Jan Claesen
Cécile Clavaud
David W. Cleary
Luis Pedro Coelho
Maureen Coleman
James Collins
Fergus Collins
Andre M. Comeau
Lydia M. Contreras
Brian K. Coombes
Kevin M. Coombs
Vaughn S. Cooper
Teresa M. Coque
Jacques Corbeil
Melissa A. Cregger
John Cryan
Christina A. Cuomo
Tom Curtis
Washington da Silva
José Freire da Silva Neto
Emily Davenport
Alan R. Davidson
Thomas Dawson
Svetlana N. Dedysh
Claire de La Serre
Manuel Delgado-Baquerizo
Laurence Delhaes
Elaine Cristina De Martinis
Alexandre de Menezes
Marjan De Mey
Haiteng Deng
Mahesh Desai
Matsapume Detcharoen
Suzanne Devkota
Willem M. de Vos
Bert Devriendt
Floyd E. Dewhirst
George C. Dicenzo
Christian Diener
César Díez-Villaseñor
Patricia A. Digiuseppe Champion
Joseph P. Dillard
Cecilia Di Ruberto
Daniel Distel
Dirk P. Dittmer
Yohei Doi
Tao G. Dong
Ann Donoghue
Rodolpho Martin do Prado
Tobias Dörr
Gavin M. Douglas
Simon L. Dove
Theo W. Dreher
Adam Driks
Alexandre Drouin
Edward G. Dudley
John Dunbar
Sylvia Duncan
Claire Duvallet
Mohammed Dwidar
Joseph Edwards
Timothy Ejike Egbo
Patrick Eichenberger
Mark A. Eiteman
Nazira El-Hage
Fadi Elias El-Rami
Bert Ely
Philipp Engel
Whitney Eileen England
Carmen Espejo
A. V. Espinel-Ingroff
Mehrbod Estaki
Alessandra S. Eustaquio
Edouard Evangelisti
Joseph O. Falkinham
Karoline Faust
Conor Feehily
Gabriel da Rocha Fernandes
Celio Fernando Figueiredo Angolini
Scott G. Filler
Steven E. Finkel
Marco Fondi
Adriana Forero
Leonard J. Foster
Farnaz Fouladi
Michael France
Kurt Fredrick
Marcelo Freire
Steven Frese
Michael W. Friedrich
Bettina C. Fries
Julia Fukuyama
Ohad Gal-Mor
Alexander Gamisch
Michael G. Ganzle
Francisco García-Del Portillo
Neha Garg
Roger Garrett
H. R. Gaskins
Josep M. Gasol
Kristi Gdanetz
Mikhail S. Gelfand
Raad Gharaibeh
Scott Michael Gifford
Douglas Paul Gladue
Laura Glendinning
Gregory B. Gloor
Erica M. Goss
Benjamin Goudey
Revathi Govind
David C. Grainger
Leigh Greathouse
Todd M. Greco
Kacy Greenhalgh
Ann Gregory
Hans J. Griesser
Robert Griffins
Maureen Groer
Mathieu Groussin
Harald R. Gruber-Vodicka
Calin Guet
Brian Haas
Elaine M. Haase
Mike Hadfield
Live H. Hagen
Matthias Hahn
Jarrad Hampton-Marcell
Kim M. Handley
Cara H. Haney
William R. Harcombe
Dennis J. Hartigan-O’Connor
Mohamed-Amine Hassani
Stijn Hawinkel
Richard Hayes
Anna Heintz-Buschart
Bernard Henrissat
Chris Henry
Markus J. Herrgård
Deborah M. Hinton
Kendal Hirschi
Thomas Hitch
Mengfei Ho
Lucas R. Hoffman
Deborah A. Hogan
Georgina Louise Hold
Nicola Holden
Hsin-Ho Huang
Jean J. Huang
Shi Huang
Yi-Xin Huo
Robert Hutkins
Muhammad Imran
Ikbal Agah Ince
Marie-Agnès Jacques
Pratik Jagtap
Laura R. Jarboe
Michael Jewett
Lin Jiang
Shuo Jiao
Jay-Hyun Jo
Rheinallt M. Jones
Susan Joseph
Sean P. Jungbluth
Fatah Kashanchi
Purna Chandra Kashyap
Justin R. Kaspar
Kevin T. Kavanagh
Daniel B. Kearns
Scott P. Keely
Scott T. Kelley
Eduard J. Kerkhoven
Megan R. Kiedrowski
Minsuk Kim
Pan-Jun Kim
Seon-Won Kim
Benjamin King
Kuniki Kino
Nichole R. Klatt
Vanja Klepac-Ceraj
Martin G. Klotz
Claudia Knief
Dan Knights
Matthew D. Koci
Uma Koduru
Arash Komeili
Heidi Kong
Konstantinos T. Konstantinidis
Omry Koren
Panagiotis Kougias
Elizabeth B. Kujawinski
Prashant Kumar
Roshan Kumar
Ranjith Kumavath
Benoit Kunath
Thomas Kuyper
Leo Lahti
Chad R. Laing
Calvin Ho-Fung Lau
Adi Lavy
Sarah Lebeer
Sarah L. Lebeis
Kyongbum Lee
Gabriela G. S. Leite
Vanessa Leone
Elisabeth Letellier
Mark Alexander Lever
Roger C. Levesque
David Levy-Booth
Ruth E. Ley
Huiying Li
Xian-Zhi Li
Xin-Di Liao
Xiaoxia Lin
Mary Lipton
John J. Lipuma
Jinhua Liu
Jinxin Liu
Yubing Liu
Kenneth J. Locey
Frank E. Löffler
Talita Lourenço
James Lowe
Tiffany Marie Lowe-Power
Sebastian Luecker
Tillmann Lueders
Diana Luise
Peter Adrian Lund
Antoni Luque
Courtney Luterbach
Michael Lyu
Wenjun Ma
Frank Michael Maixner
Thulani P. Makhalanyane
Antoine Malabirade
Antonino Malacrinó
Anthony Malanoski
Rex R. Malmstrom
Ashutosh K. Mangalama
Sam Manna
Fei Mao
Maria L. Marco
Christopher W. Marshall
Philippe Marteau
Willm Martens-Habbena
Jose-Luis Martinez-Gonzalez
Fumito Maruyama
Norman Mauder
Meghan May
Andrew J. Mcbain
Cameron McBride
Mark J. McBride
Jessica R. McCann
Ryan McClure
Diane McDougald
Andrew McDowell
Elizabeth A. McGraw
K. Kai McKinstry
Sandra L. McLellan
Katherine McMahon
Marnix H. Medema
Carlos Medina
Peter Meinicke
Alessio Mengoni
Ilhem Messaoudi
Julie Meyer
Kate Michel
Yusuke Minato
Aaron P. Mitchell
Masatoshi Miyakoshi
Jennifer M. Mobberley
Andrew Moeller
Jonathan Monk
James J. Moran
Jacob Moran Gilad
Robert M. Morris
Douglas Morrison
Rafal Mostowy
Vladimir L. Motin
Patricia Mowery
Maitreyee Mukherjee
Emilie E. L. Muller
Delia Muntean
Marc Mussmann
Jay Nadeu
Nikhil Nair
Yuji Naito
Teru Nakatsuji
Fernando Navarro-Garcia
Dipti D. Nayak
Julia Neilson
Tiffanie Nelson
Jeniel E. Nett
Markus Nett
Anthony P. Neumann
Graeme William Nicol
Henrik Nilsson
Aleksandra Nita-Lazar
Justin R. Nodwell
Teresa Nogueira
J. Staffan Normark
Spencer V. Nyholm
Paul Alexander O’Brien
Mary O’Connell-Motherway
Pierre Offre
Andrew Ogram
Niall O’Leary
Matthew R. Olm
Randall J. Olsen
Michelle Ann O’Malley
Dana Opulente
Anne-Marie Overstreet
Egon Anderson Ozer
Robert J. Palmer
Gianni Panagiotou
Dane Parker
Raghuveer Parthasarathy
Alessandro Passera
Rob Patro
Sushmita Patwardhan
Talima Pearson
Philip E. Pellett
Beatriz Penalver
Hong-Juan Peng
Rita C. Pessotti
Marie-Agnes Petit
Daniel Petras
Jennifer Pett-Ridge
Jody Phelan
Vanessa Phelan
Harold Pimentel
Azul Pinochet-Barros
Constanze Pinske
Lucía Pita
Thomas G. Platt
Patrick Plésiat
Przemyslaw Plocinski
Georg Pohnert
Argyris Politis
Alicia Ponte-Sucre
Hannah Beth Pooley
Phillip B. Pope
Steven L. Porter
Sambhawa Priya
Nan Qin
Wensheng Qin
Zhe-Xue Quan
Robert Andrew Quinn
Korneel Rabaey
Mirjana Rajilić-Stojanović
Thandavarayan Ramamurthy
Kelly Ramirez
Giordano Rampioni
Matthew Mark Ramsey
Lutgarde Raskin
Philip N. Rather
Thomas Rattei
Gregor Reid
Olaya Rendueles Garcia
Federico Rey
Peter Richard
Miles Richardson
Lionel Rigottier-Gois
Michael Scott Robeson
Jennifer Rocca
Jeremy Rock
Kyle H. Rohde
Caroline Roper
David A. Rosen
Ashley A. Ross
Simon Roux
Maxim Rubin Blum
Luis A. Rubio
Kelly V. Ruggles
Thomas A. Russo
Maurizio Ruzzi
Sabrina Sabo
Zakee L. Sabree
Jon Sanders
Haley Sanderson
Todd Sandrin
Alyson Santoro
Guillaume Sarrabayrouse
Brandon Satinsky
Jimmy Saw
Lizbeth Sayavedra
Joy Scaria
Mark A. Schembri
Kurt Schesser
Joshua P. Schimel
Amy K. Schmid
Anthony P. Schmitt
Lars Schreiber
Matthew O. Schrenk
H. Steven Seifert
Adnane Sellam
Aditi Sengupta
Rushina Shah
Robert M. Q. Shanks
Lori R. Shapiro
Ashok Kumar Sharma
Xinlei Sheng
Liat Shenhav
Francesca L. Short
Mark W. Silby
Justin D. Silverman
Andrew Simpson
Mitchell Singer
Steven Singer
Rohita Sinha
Timofey Skvortsov
Beate M. Slaby
Barth F. Smets
Daniel P. Smith
David G. E. Smith
Kim Sneppen
Lindsay Solden
Deguang Song
Jiangning Song
Eva C. Sonnenschein
Utkarsh Sood
Jorg Soppa
Anja Spang
Stephen Spiro
Joseph E. Spraker
Jason E. Stajich
Laura Steindler
George C. Stewart
Francesco Strati
Reed Studdendieck
Xiaoquan Su
David Sue
Hikaru Suenaga
Zheng Sun
Mehul Suthar
Yasuhiro Suzuki
Wesley D. Swingley
Jason B. Sylvan
Ilias Tagkopoulos
Guylaine Talbot
Yinjie J. Tang
Gerald William Tannock
Shengce Tao
W. Andy Tao
Jaclyn N. Taroni
Bradford P. Taylor
Ben Temperton
Benno Herman Ter Kuile
Luke R. Thompson
Yun Tian
Laura Tipton
Mehdi Toghi
Maya Topf
Tamas Torok
Gloria Torres-Cortes
Carolina Tropini
Benjamin John Tully
Silvia Turroni
Carles Ubeda
Beatrix M. Ueberheide
Sabah Ul-Hasan
Alex van Belkum
Petra Van Damme
Carin K. Vanderpool
Tom Van de Wiele
Willem van Schaik
Douwe van Sinderen
Luciana Vasquez-Pinto
Tommi Vatanen
Yoshiki Vazquez-Baeza
Siddarth Venkatesh
Marie-Joelle Virolle
Chris Voigt
Robin Voigt-Zuwala
Joseph Thomas Wade
Irene Wagner-Dobler
Alan W. Walker
Matthew Wallenstein
Guanghua Wang
Jerry Wang
Joyce Wang
Wei Wang
Xuya Wang
Yaqiang Wang
Zeneng Wang
Zhang Wang
Doyle Ward
Wei Wei
Margaret Weinroth
Martin Welker
Nicole Wheeler
Sean P. J. Whelan
Christopher Whidbey
Marvin Whiteley
Anisha Wijeyesekera
Roland Conrad Wilhelm
Roli Wilhelm
Amy D. Willis
Tomasz Wilmanski
Jennifer R. Wilson
Jesse Wilson
Jeffrey H. Withey
Benjamin Woolston
Erik S. Wright
Rosanna C. T. Wright
Fuqing Wu
Hao Wu
Michael Wu
Min Wu
Yu-Wei Wu
Todd N. Wylie
Kelly Wyres
Guoxiang Xie
Jiatao Xie
Chunlan Xu
Peng Xu
Xuewei Xu
Zhenjiang Zech Xu
Ching-Hong Yang
Filiz Yarimcam Saglam
Alyson L. Yee
V. Laxmi R. Yeruva
Yibing Yin
Yasuo Yoshida
Jun Yu
Xiaoqian Yu
Zhongtang Yu
Mengting Yuan
Yuanchao Zhan
Changyi Zhang
Faming Zhang
Jiachao Zhang
Kai Zhang
Limin Zhang
Quan-Guo Zhang
Weipeng Zhang
Zhengguang Zhang
Ziding Zhang
Fangqing Zhao
Rui Zhao
Shijie Zhao
Xin-Qing Zhao
Dongsheng Zhou
Wenhao Zhou
Liangquan Zhu
Yan Zhu
Jun Zou

